# Asymmetry in neurovascular bundle blood flow in prostate cancer patients: A pre‐treatment doppler ultrasound study

**DOI:** 10.1002/acm2.70211

**Published:** 2025-08-24

**Authors:** Jing Wang, Xiaofeng Yang, Boran Zhou, James J. Sohn, Richard L. J. Qiu, Pretesh Patel, Ashesh B. Jani, Tian Liu

**Affiliations:** ^1^ Department of Radiation Oncology Icahn School of Medicine at Mount Sinai New York New York USA; ^2^ Department of Radiation Oncology and Winship Cancer Institute Emory University Atlanta Georgia USA; ^3^ Department of Radiation Oncology MedStar Georgetown University Hospital Washington District of Columbia USA; ^4^ Department of Radiation and Cellular Oncology The University of Chicago Chicago Illinois USA

**Keywords:** blood flow, doppler ultrasound, neurovascular bundles (NVBs), prostate cancer, radiotherapy, sexual function

## Abstract

**Background:**

Neurovascular‐sparing treatment is believed to help preserve erectile function for localized prostate cancer, given the key role of the arterial supply of neurovascular bundles (NVBs) in potency recovery post‐treatment. While NVB‐sparing radiotherapy (RT) is emerging, imaging methods to assess NVB function are lacking.

**Purpose:**

This study aims to evaluate the functional status of bilateral NVBs using pulsed wave Doppler ultrasound in patients undergoing prostate RT.

**Methods:**

Fifty‐seven patients (mean age: 66.2 ± 7.1 years) were enrolled in this single‐institute prospective study. Each patient underwent a transrectal ultrasound scan in the lithotomy position. Bilateral blood flow in the NVBs was measured using pulsed wave Doppler ultrasound. A custom program was developed to automatically detect and analyze the Doppler spectral waveform. Five Doppler parameters were extracted: peak systolic velocity (PSV), end‐diastolic velocity (EDV), mean velocity (Vm), resistive index (RI), and pulsatile index (PI). Discrepancies in Doppler parameters between the left and right sides were calculated. Patient‐reported sexual outcomes were assessed using the Expanded Prostate Cancer Index Composite for Clinical Practice (EPIC‐CP).

**Results:**

The Doppler pulsed waveform parameters for the 57 patients were: PSV = 11.0 ± 4.0 cm/s, EDV = 1.3 ± 1.9 cm/s, Vm = 4.0 ± 2.4 cm/s, RI = 0.89 ± 0.14, and PI = 3.46 ± 1.80. Analysis of PSV revealed differing blood flow between the left and right NVBs: 40 patients had <50% difference, 10 patients had a 50%–100% difference, and seven patients had >100% difference. Among patients aged 65 years or younger (*n* = 11) with EPIC‐CP scores, blood flow was negatively correlated to erectile dysfunction (Spearman correlation coefficient of −0.71, *p* = 0.01).

**Conclusions:**

Substantial differences in blood flow between bilateral NVBs were observed. The functional information obtained from NVB Doppler ultrasound may be valuable in guiding individualized NVB‐sparing treatment planning.

## 1 INTRODUCTION

Sexual potency impairment is a common complication resulting from radiotherapy (RT) for prostate cancer, affecting a large proportion of patients.^1^, ^2^ Clinical studies have shown that approximately 38%–62% of men will experience radiation‐induced erectile dysfunction (ED).^1^, ^3^, ^4^, ^5^ Despite its prevalence, the precise mechanisms underlying radiation‐induced ED remain controversial. Since early 2010, the penile bulb has been identified as an organ at risk for prostate RT.^6^, ^7^ However, the relationship between the dose received by the penile bulb and the incidence of ED has been a subject of ongoing debate.^8^, ^9^ Some studies have proposed that the dose to the penile bulb can predict the development of radiation‐induced ED.^6^, ^7^, ^10^ Conversely, other studies have found no correlation between the mean dose to the penile bulb and post‐treatment ED.^11^, ^12^, ^13^

The bilateral neurovascular bundles (NVBs), situated along the postero‐lateral aspect of the prostate, are increasingly recognized as potential critical structures in the development of ED.^14^, ^15^, ^16^ While NVB preservation has become a standard of care in radical prostatectomy for patients diagnosed with localized prostate cancer,^17^ it is not yet a standard consideration in prostate RT protocols. Recently, studies have demonstrated both the reproducibility of NVB contouring using MR images^18^ and the feasibility of NVB‐sparing external beam RT (EBRT) in treatment planning.^19^ Additionally, a feasibility study utilizing MRI‐guided adaptive radiotherapy suggested that nearly 50% of prostate cancer patients could be eligible for NVB‐sparing treatment.^20^ This study found that the NVBs could potentially be spared bilaterally in 20.0% and unilaterally in 68.0% of patients. Despite these promising findings, there is a noticeable lack of imaging studies specifically focusing on the NVB or RT‐induced NVB injury.^21^ This highlights the need for advanced imaging modalities that can facilitate the routine incorporation of NVB sparing into treatment planning processes.

Doppler ultrasound, using clinically accepted parameters such as peak systolic velocity (PSV), end‐diastolic velocity (EDV), and resistive index (RI), provides valuable insights into vascular health status.^22^, ^23^ Previous research has explored utilizing Doppler ultrasound velocity waveform features for quantitative assessments of arterial blood flow.^24^, ^25^, ^26^, ^27^, ^28^ However, investigations into NVB blood flow are limited, with the majority of studies conducted in the context of prostate biopsy or surgical settings.^29^, ^30^ Comprehensive studies examining NVB blood flow in the context of prostate RT are particularly scarce.

In this study, we have conducted a Doppler ultrasound imaging study to evaluate NVB blood flow in patients who opt for high‐dose‐rate (HDR) brachytherapy, with or without EBRT, for prostate cancer. We aim to contribute to the refinement of imaging methodologies for NVB assessment with pulsed wave Doppler technology. Pulsed wave Doppler may offer physicians valuable insights into the vascular flow in the NVBs, which can be instrumental in enhancing unilateral or bilateral NVB‐sparing treatment strategies for prostate cancer patients. The central hypothesis is that Doppler ultrasound can assess baseline NVB function and support informed side selection for NVB preservation. All Doppler measurements were acquired prior to the initiation of any radiotherapy, ensuring the data reflect true pre‐treatment conditions. These measurements revealed baseline asymmetries in NVB blood flow and allowed us to explore correlations between Doppler parameters and pre‐existing erectile function, particularly in younger patients who are more likely to benefit from NVB‐sparing interventions.^31^ Together, these findings aim to provide functional imaging evidence that can complement anatomical considerations when determining whether, and on which side, NVB sparing is clinically warranted.

## 2 METHODS

### 2.1 Patient study–doppler ultrasound of the NVBs

**FIGURE 1:**
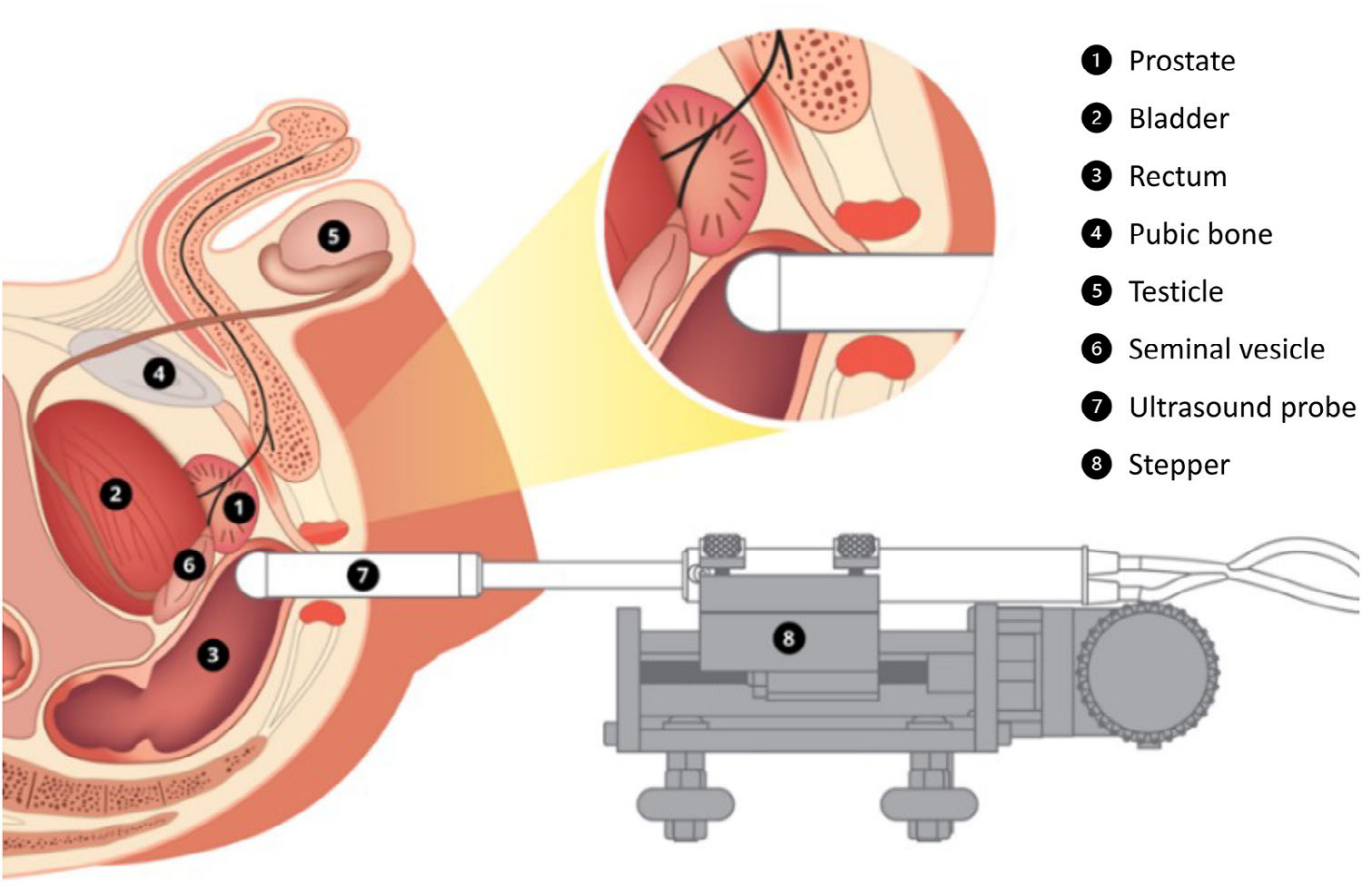
Diagram illustrating B‐mode and Doppler ultrasound scans. The patient is scanned in a lithotomy position using a probe. The probe is held with the mechanical stepper.

Under an institutional review board‐approved protocol, patients consented to this single‐institute prospective ultrasound study. Each patient underwent a transrectal ultrasound scan of the prostate, along with Doppler measurement of the NVBs. All NVB Doppler measurements were performed during the HDR brachytherapy procedure, prior to the delivery of any radiation therapy. As such, this cohort represents true pre‐radiotherapy conditions, free from any radiation‐induced changes. As illustrated in Figure 1, patients were scanned in the lithotomy position with an empty bladder, following the standard prostate ultrasound protocol for fiducial marker placement and brachytherapy.

**FIGURE 2:**
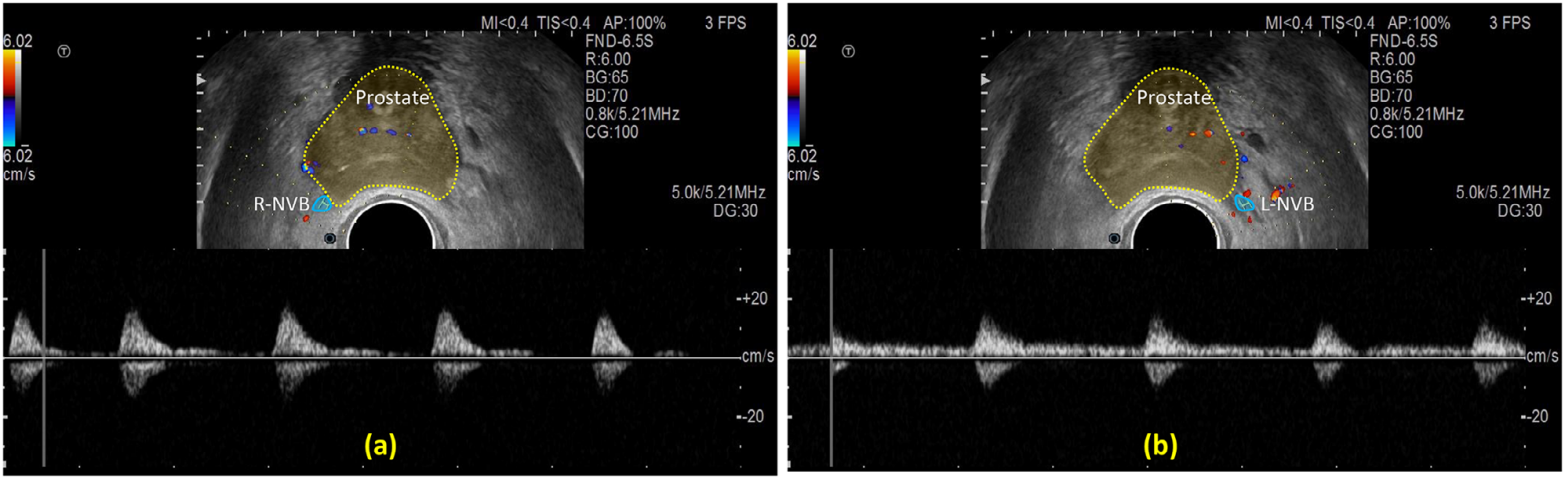
An example of a patient's pulsed wave Doppler measurements of (a) right and (b) left NVBs. The prostate is outlined with a dotted yellow line, and the right and left NVBs are outlined with blue lines in the ultrasound images. The pulsed wave Doppler signals are below the ultrasound images.

A conventional ultrasound scanner (HI VISION Avius, Hitachi Medical Group, Japan) equipped with a 7.5 MHz bi‐plane probe (EUP‐U533C) was used in this study. The probe was securely mounted with a mechanical stepper (Bard Medical, Inc., Covington, GA). Utilizing the stepper, the radiation oncologist maneuvered the probe to perform a B‐mode scan of the entire prostate gland, capturing images from the base to the apex in the axial view, and rotating the probe between the left and right sides in the longitudinal view. We optimized and fixed the ultrasound setting to ensure standardized scanning among all patients. The ultrasound scanning used the following settings for B‐mode visualization: a 7.5‐MHz center frequency, a focal length of 3.3 cm, a depth of 5 cm, a brightness gain of 65, and a frame rate of 18 frames/s. Color Flow Doppler displays blood‐velocity signals on the full screen, allowing clinicians to identify the location where NVB signals are most clearly visualized, typically around the mid‐prostate. Once this plane is located, the probe is secured in the stepper, and Pulsed wave Doppler measurements are acquired for NVB sites. The default Doppler settings: central frequency of 5.21 MHz, pulse repetition frequency of 5.0 k, Doppler gain of 30, gate size (sample volume) of 2 mm, angle of 0°, and wall filter of 84. For Pulsed wave Doppler measurements, it is important to address the angle dependence in velocity values, for example, PSV, EDV, and Vm. Instead of characterizing the angle between the doppler beam and the NVB arterial direction for each patient, we used a fixed angle of 0° throughout all measurements. Although individual anatomical variations exist, the left and right NVBs generally follow similar anatomical pathways. All Doppler measurements were taken near mid‐prostate location, with the ultrasound beam aligned to capture the strongest possible blood flow signal, helping to ensure reasonable angle consistency across the cohort. Figure 2 presents an example ultrasound of the prostate gland, highlighting the posteriorlateral NVBs on both the left and right sides, along with the pulsed wave Doppler signals acquired from each side.

### 2.2 Pulsed wave doppler waveform extraction and parameter analysis

While conventional ultrasound systems report Doppler parameters such as PSV, EDV, and RI, they can be unreliable in the presence of spectral noise. To improve accuracy, we developed a custom automated spectral waveform analysis tool, written in Python (version 3.7.15). This program automatically identifies and analyzes the Doppler spectral waveform, providing more robust and consistent assessment of key flow parameters.

#### 2.2.1 Preprocessing steps

The preprocessing of the Doppler signal involved several crucial steps to enhance the visibility and accuracy of waveform extraction: (1) Renormalization: The Doppler signal was renormalized to enhance the brightness of the pixels, providing a clearer and more distinct view of the Doppler waveform; (2) Intensity Thresholding: An intensity thresholding technique was applied at 40% of the maximum brightness to identify the pixel boundaries of the Doppler spectra, ensuring precise delineation of the waveform; and (3) Spectral Smoothing: A Gaussian filter was applied to smooth the spectral data, reducing noise and improving the clarity of the Doppler waveform. Details of preprocessing steps are provided in the Supplementary materials.

#### 2.2.2 Feature extraction

After preprocessing, five Doppler features were recorded to characterize the blood flow within the NVB. Three key velocity (cm/s) features were extracted from the waveform, including PSV, EDV, and mean velocity (Vm). In addition to these features, two dimensionless indices were calculated: resistive index (RI) and pulsatility index (PI):

(1) $$\mathrm{RI}=\frac{\mathrm{PSV}\text{--}\mathrm{EDV}}{\mathrm{PSV}}$$

(2) $$\mathrm{PI}=\frac{\mathrm{PSV}\text{--}\mathrm{EDV}}{\mathrm{Vm}}$$

To evaluate discrepancies in bilateral blood flow, the percentage difference between these Doppler features on the left and right sides was calculated as the bilateral discrepancy:

(3) $$\mathrm{Bilateral}\;\mathrm{discrepancy}=\frac{\mathrm{Max}\text{--}\mathrm{Min}}{\mathrm{Min}}\times 100\%$$

where Max and Min are the greater and lesser values between the left and right NVBs, respectively.

### 2.3 Sexual function assessment

Patients’ sexual function was assessed using the expanded prostate cancer index composite for clinical practice (EPIC‐CP) questionnaire.^32^ This validated instrument allows patients to self‐report various aspects of quality‐of‐life including sexual health. The erectile function score was calculated from the responses to three targeted sexual symptom questions:

1. How would you rate your ability to reach orgasm (climax)? 0: Very good; 1: Good; 2: Fair; 3: Poor; and 4: Very poor to none.
2. How would you describe the usual quality of your erections? 0: Firm enough for intercourse; 1: Firm enough for masturbation and foreplay only; 2: Not firm enough for any sexual activity; and 4: None at all.
3. Overall, how much of a problem has your sexual function or lack of sexual function been for you? 0: No problem; 1: Very small problem; 2: Small problem; 3: Moderate problem; and 4: Big problem.

The scores are summed to produce a total erectile function score, which ranges from 0 to 12. Higher scores indicate greater severity of ED: 9 or more (high severity), 5–8 (moderate), and 1–4 (low severity).^33^

The erectile function scores were analyzed in conjunction with the Doppler ultrasound parameters to evaluate the potential correlation between the erectile function and Doppler measurements of the bilateral NVB function.

### 2.4 Generative AI and large language models declaration

AI tools (Chat‐GPT) were used to help prepare this manuscript.

## 3 RESULTS

### 3.1 Patient results

From 2021 to 2023, 57 patients (66.2 ± 7.1 years) scheduled to receive prostate HDR were enrolled. Patient and treatment characteristics are detailed in Table 1. All imaging data were acquired during the HDR procedure, specifically after the anesthesia and before the needle insertion. A radiation oncologist performed all the imaging acquisition. For all patients, the left and right NVBs were successfully identified using color Doppler followed by pulsed wave Doppler measurements of the blood flow. The entire Dopper measurement takes less than 5 min.

**TABLE 1:** Patient characteristics (*n* = 57).

Characteristics	Value
Age (y)	
Mean ± std	66.2 ± 7.1
Race	
African American	30
White	27
EPIC‐CP score^a^ (n = 26)	
0	10
1–4	6
5–8	6
9–12	4
Patients’ Gleason grade (prostate cancer)	
6 (3 + 3)	4
7 (3 + 4)	31
7 (4 + 3)	6
8 (3 + 5)	1
8 (4 + 4)	6
9 (4 + 5)	8
9 (5 + 4)	1

^a^ Expanded prostate cancer index composite for clinical practice (EPIC‐CP). Only 26 patients self‐reported these scores, with higher scores indicate greater severity of erectile dysfunction: 9 or more (high severity), 5–8 (moderate), and 1–4 (low severity).

**FIGURE 3:**
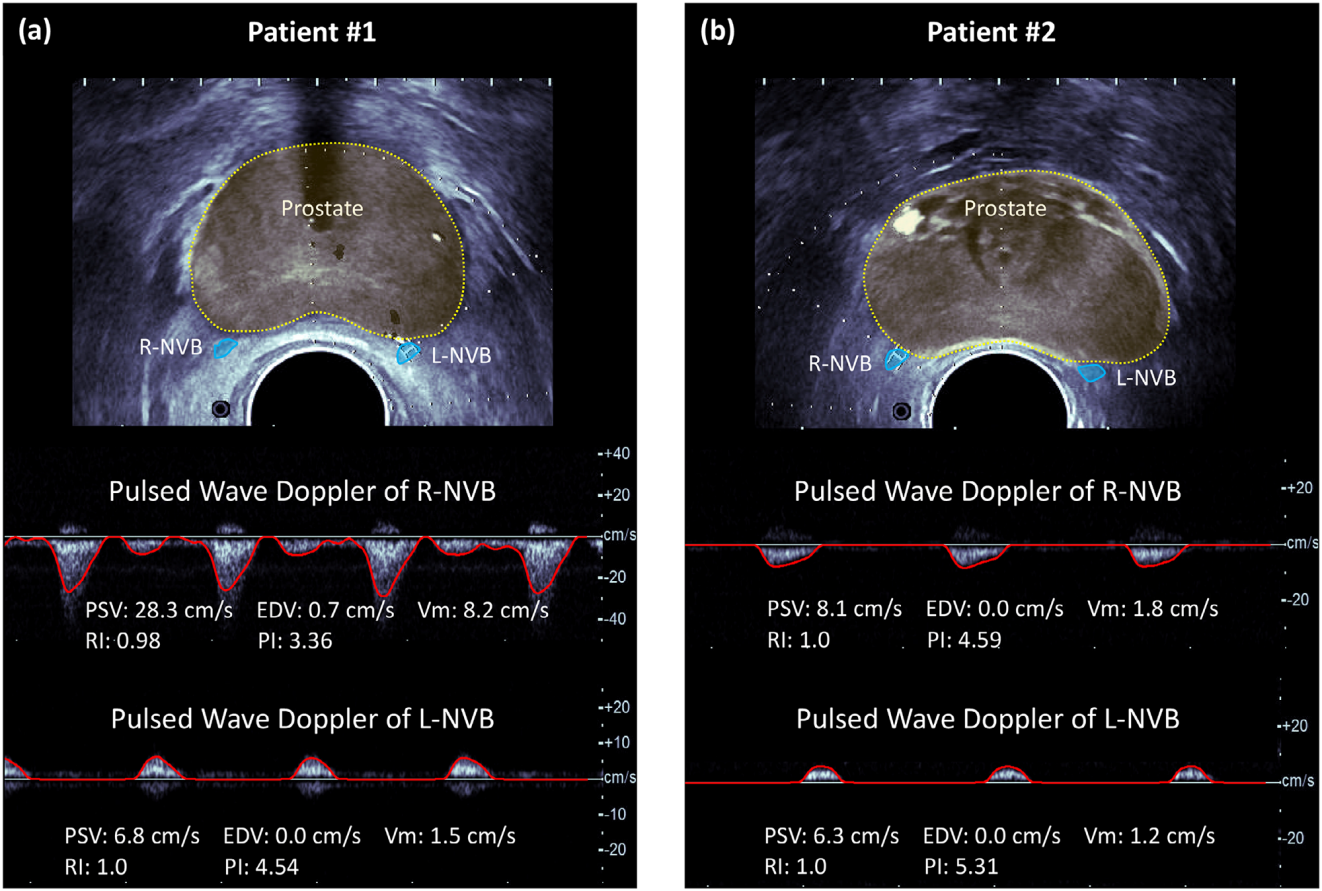
Ultrasound measurements for two patients: (a) patient #1 with normal erectile function (EPIC‐CP score = 0) and (b) patient #2 with moderate ED (EPIC‐CP score = 6). The upper panels show B‐mode images of the prostate gland with NVBs highlighted on the right (R‐NVB).

Figure 3 illustrates two cases of Doppler measurements of the NVBs. Patient #1 is a 51‐year‐old man with prostate cancer (T‐stage 8, GS 4 + 3, PSA 71.14). At the time of Doppler acquisition, he had not received RT and his EPIC‐CP score was 0, indicating no dysfunction. His Doppler measurements were as follows: PSV = 28.3 cm/s and 6.8 cm/s, EDV = 0.7 cm/s and 0 cm/s, Vm = 8.2 cm/s and 1.5 cm/s, RI = 0.98 and 1.0, and PI = 3.36 and 4.54 for the right and left NVBs, respectively. Patient #2 is a 56‐year‐old man with prostate cancer (T‐stage 4, GS 3 + 4, PSA 6.14). Similar to Patient #1, he had not received RT at the time of Doppler measurement. His EPIC‐CP score was 6, indicating moderate ED. His Doppler measurements were as follows: PSV = 8.1 cm/s and 6.3 cm/s, EDV = 0 cm/s and 0 cm/s, Vm = 1.8 cm/s and 1.2 cm/s, RI = 1.0 and 1.0, and PI = 4.59 and 5.31 for the right and left NVBs, respectively.

For all 57 patients, the five pulsed wave Doppler parameters determined from waveforms are as follows: PSV = median 10.1, range 4.8–34.9 cm/s, EDV = median 0 cm/s, range 0–12.2 cm/s, Vm = median 3.2 cm/s, range 0.9–17.7 cm/s, RI = median 1.0, range 0.27–1.0, and PI = median 3.17, range 0.39–11.69. Moreover, between left and right NVBs, large variations in the bilateral discrepancy of Doppler parameters were observed (Table 2). For PSV values, 40 patients had less than 50% difference, 10 patients had 50%–100% difference, and seven patients had over 100% difference.

**TABLE 2:** Descriptive statistics of the five pulsed wave Doppler parameters and their bilateral discrepancies between left and right NVBs (*n* = 57).

	PSV	EDV	Vm	RI	PI
Mean value	11.0 ± 4.0 cm/s	1.3 ± 1.9 cm/s	4.0 ± 2.4 cm/s	0.89 ± 0.14	3.46 ± 1.80
Range	4.8–34.9 cm/s	0–12.2 cm/s	0.9–17.7 cm/s	0.27–1.00	0.39–11.69
Bilateral discrepancy	48 ± 56%	880 ± 1025%	85 ± 106%	27 ± 48%	101 ± 128%

The percentage discrepancy for EDV was calculated based on 14 patients, excluding those with an EDV value of 0.

### 3.2 Correlation between doppler parameters and sexual function

Among the 57 enrolled patients, sexual function assessment using the EPIC‐CP questionnaire was available for 26 subjects (mean age 67 ± 8, range 51–83 years). In the younger group (age ≤ 65 years, 11 patients), we found a significant negative correlation between the mean PSV value of the left and right sides and the EPIC‐CP score, with a Spearman correlation coefficient of −0.71 (*p* = 0.01). In contrast, in the older group (age > 65 years, 15 patients), no pulsed wave Doppler parameter showed a significant correlation with the erectile score (*p* < 0.05), indicating the erectile function in older group is more complex and multi‐factorial, consistent with previous study.^10^ The age division of younger (≤65 y) and older (>65 y) patients followed the previous literatures^10^, ^31^ studying erectile dysfunction in prostate cancer patients. Moreover, no significant differences were observed in the five Doppler features between the younger (≤65 years) and older (>65 years) groups: PSV = 9.8 ± 3.1 and 9.8 ± 2.4 cm/s (*p* = 0.99), EDV = 1.2 ± 1.6 and 0.8 ± 1.2 cm/s (*p* = 0.45), Vm = 3.8 ± 1.8 and 3.3 ± 1.6 cm/s (*p* = 0.50), RI = 0.87 ± 0.17 and 0.91 ± 0.12 (*p* = 0.46), and PI = 2.26 ± 1.72 and 3.44 ± 1.48 (*p* = 0.77) for the younger and older groups, respectively, using a student's *t*‐test assuming two‐sample unequal variance. Due to the limited sample size, these results only refuted very large differences between younger and older groups. A larger dataset is needed to detect more subtle differences.

### 3.3 Power analysis for sample size

With 57 paired observations, the study had 80% power to detect a standardized bilateral difference of Cohen's *d* = 0.53. The observed Cohen's d values between the greater and lesser sides of bilateral Doppler parameters are 0.88, 0.74, 0.73, 0.84, and 0.79 for PSV, EDV, Vm, PI, and RI, respectively. All are well above the detection threshold, confirming sufficient power for our primary objective of characterizing left–right asymmetry.

For the correlation analysis in the younger cohort, we aimed to detect a large, biologically meaningful association (|ρ| ≈ 0.7). With *n* = 11, the study had approximately 71% power (Fisher z‐transformation) to detect a correlation of this magnitude. The observed correlation reached statistical significance, indicating acceptable sensitivity for our exploratory functional hypothesis.

The age‐group comparison was also exploratory. Among participants with EPIC‐CP scores, the age distribution (11 aged ≤ 65 vs. 15 aged > 65) provided 80% power only to detect large effect sizes (*d* > 1.1). The observed Cohen's *d* values for the five Doppler parameters—0.007, 0.31, 0.28, 0.31, and 0.12 for PSV, EDV, Vm, PI, and RI, respectively—suggested that any true effect is likely small. Therefore, the absence of significant differences between age groups should be interpreted with caution: while large differences were ruled out, the study was not powered to exclude medium or small effects.

## 4 DISCUSSION

This study used pulsed wave Doppler to investigate NVB blood flow in patients receiving prostate RT, representing the largest cohort studied to date. By examining Doppler waveform parameters such as PSV, EDV, Vm, RI, and PI, our findings offer valuable insights into the functional assessment of NVBs. Despite the crucial role of NVBs in preserving sexual function during prostate cancer treatment, few studies have measured their blood flow. Leventis et al. reported NVB RI values around 0.98 in a Doppler ultrasound study.^34^ In the largest prostate biopsy cohort, Tsai et al. used a fixed isonation angle of 56° for all measurements and reported mean PSV values ranging from 20–25 cm/s, mean EDVs from 4 to 7 cm/s, and mean RIs between 0.7 and 0.8 in both benign and malignant cases.^29^ In contrast, our study did not apply angle correction, as the true angle of insonation could not be reliably determined in the intraoperative setting. All Doppler acquisitions were performed using a standardized approach, with the ultrasound beam aligned to capture the strongest possible blood flow signal, but without specifying or correcting for an insonation angle. Consequently, our measured velocity values (mean PSV: 11.0 cm/s; mean EDV: 1.3 cm/s) are lower than those reported in angle‐corrected studies. Nonetheless, angle‐independent indices such as RI (mean: 0.89) and PI remain robust to angular variation and support the validity of intra‐cohort comparisons in our analysis.

Using PSV values as the key indicator of blood flow, we observed mean bilateral discrepancies as large as 48 ± 56% across the cohort. Notably, around 30% of the cohort exhibited discrepancies greater than 50%. These bilateral discrepancies in NVB blood flow highlight the complexity of vascular dynamics in the prostate region, with implications for treatment planning and outcomes in patients undergoing prostate RT. Several studies have demonstrated the dosimetric feasibility of NVB‐sparing RT.^19^ Teunissen FR et al. reported that NVBs could be spared bilaterally in 20.0% and unilaterally in 68.0% of patients eligible for NVB‐sparing RT.^20^ When unilateral NVB sparing is considered, tumor location remains the primary factor dictating which side may be safely spared. In cases where either side can be preserved without compromising tumor control, Doppler ultrasound provides quantitative assessment of side‐specific blood flow, revealing substantial inter‐lateral differences. This functional information may assist in selecting the better‐perfused bundle for preservation, with the goal of optimizing erectile function while maintaining adequate tumor coverage.

Moreover, the correlation analysis between pulsed wave Doppler parameters and sexual function scores adds another dimension to the study findings. In this study, we examined a cohort of patients aged 65 years or younger (*n* = 11) and confirmed the correlation between decreased PSV values and erectile dysfunction (Spearman correlation coefficient = −0.71, *p* = 0.01). Although the hypothesis that a decrease in NVB blood flow correlates with erectile dysfunction is preliminary and intuitive, both the previous longitudinal case study^35^ and this cohort study suggest a potential link between NVB Doppler parameters and sexual function outcomes. These findings highlight the importance of considering vascular health in treatment decision‐making and underscore the potential role of pulsed wave Doppler as a tool for managing sexual function outcomes in this patient population.

The findings of this study have several clinical implications. The observed variations in pulsed wave Doppler parameters between bilateral NVBs confirm the importance of individualized treatment planning in prostate RT. Clinicians should consider the functional status of NVBs alongside anatomical considerations to optimize treatment outcomes, particularly in preserving sexual function. Additionally, the correlation between Doppler parameters and sexual function scores, particularly for younger patients, suggests that Doppler ultrasound may serve as a non‐invasive tool for assessing vascular health and monitoring radiation‐induced NVB damage and erectile function outcomes.^35^ Integrating Doppler ultrasound into routine clinical practice could facilitate personalized treatment strategies and improve patient outcomes in prostate cancer management. For example, real‐time Doppler ultrasound can be used to identify the NVB during prostate brachytherapy. Real‐time optimization during brachytherapy planning allows an opportunity to limit the dose to unilateral or bilateral NVBs based on pulsed wave Doppler findings and potentially decrease erectile side effects.

Despite the valuable insights provided by this study, several limitations warrant consideration. The patient cohort size, particularly in the subset of patients with sexual function data, is relatively small, limiting the generalizability of the findings. Future studies with larger cohorts are needed to validate the observed correlations and explore potential confounding factors. Additionally, the study primarily focused on Doppler waveform analysis, and other imaging modalities such as MRI were not utilized for comprehensive assessment. Integrating multimodality imaging approaches could provide a more comprehensive understanding of NVB function and its implications for treatment outcomes.

Furthermore, while pulsed wave Doppler ultrasound shows promise as a tool for assessing NVB function, its operator dependency and susceptibility to probe orientation pose challenges. Future research should focus on standardizing imaging protocols and techniques to enhance its reproducibility and reliability. Additionally, longitudinal studies are needed to assess the predictive value of pulsed wave Doppler in relation to long‐term treatment outcomes and patient quality of life.

## 5 CONCLUSIONS

This study highlights the utility of pulsed wave Doppler ultrasound in evaluating the functional status of NVBs in prostate cancer patients undergoing RT. The substantial discrepancies in blood flow between bilateral NVBs and the potential correlation between PSV and erectile function in younger patients emphasize the importance of personalized NVB‐sparing RT planning. By providing insights into vascular dynamics and their correlation with sexual function outcomes, Doppler ultrasound has the potential to inform personalized RT‐sparing strategies and monitor vascular health before and after treatment. Future research should focus on larger cohorts, validate the findings, and establish Doppler ultrasound as a valuable tool in the clinical management of prostate cancer.

## AUTHOR CONTRIBUTIONS

*Conceptualization*: Tian Liu, Xiaofeng Yang. *Original writing*: Jing Wang. *Resources & Data*: Jing Wang, Pretesh Patel, Ashesh B. Jani. *Supervision*: Tian Liu. *Writing—Review & Editing*: Xiaofeng Yang, Boran Zhou, James J. Sohn, Richard L.J. Qiu, Tian Liu. *Validation*: Pretesh Patel, Ashesh B. Jani.

## CONFLICT OF INTEREST STATEMENT

The authors have no relevant conflicts of interest to disclose.

## Supporting information



Supporting information

## Data Availability

Data are available upon request from the corresponding author.
