# Supplementary material for: Asymmetry in neurovascular bundle blood flow in prostate cancer patients: A pre‐treatment doppler ultrasound study
Source: J Appl Clin Med Phys. 2025 Aug 24;26(9):e70211. doi: 10.1002/acm2.70211 (PMC12375689; doi:10.1002/acm2.70211)
Supplement: Supplementary file 1 — Supporting information [file ACM2-26-e70211-s001.docx]

**Supplementary Materials**

We designed an automated preprocessing pipeline that systematically extracts Doppler spectral waveforms. The preprocessing consists of three key steps:

**Step 1 – Renormalization.**
The raw pulsed-wave Doppler signals display substantial variation in absolute pixel intensity, precluding the use of a single intensity threshold for waveform extraction across all acquisitions. We therefore rescaled pixel intensities to a unified range, enhancing brightness and improving the definition of the waveform. Figure S-1 illustrates the renormalization effect on representative “low-intensity,” “moderate-intensity,” and “high-intensity” raw images: after renormalization, their overall brightness are comparable.


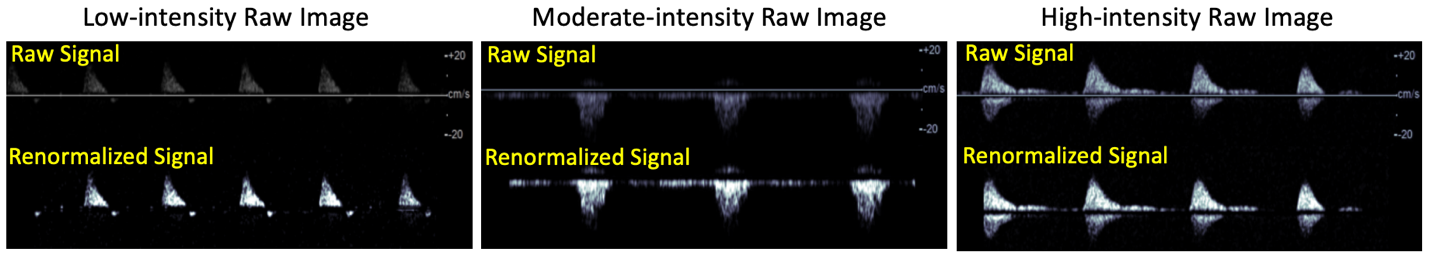


**Figure S-1.** Illustrative examples of Doppler signal renormalization.

**Step 2 – Intensity thresholding.**
To delineate the spectral envelope, we treated each time bin as a vertical column. Starting at the mid-baseline, we advanced upward or downward until the average pixel intensity within the column dropped below 40 % of the renormalized maximum; that uppermost pixel defined the velocity value for that column.

- **Low thresholds** admit speckle and noise, falsely elevating velocities (Fig. S-2, case 1).
- **High thresholds** retain only the brightest core, truncating the apex and underestimating peak values (Fig. S-2, case 3).
- In most scans the extracted waveform is robust across thresholds (Fig. S-2, case 2).

Empirical evaluation demonstrated that a 40 % intensity threshold provided the optimal trade-off between noise suppression and preservation of clinically relevant signal across the cohort.


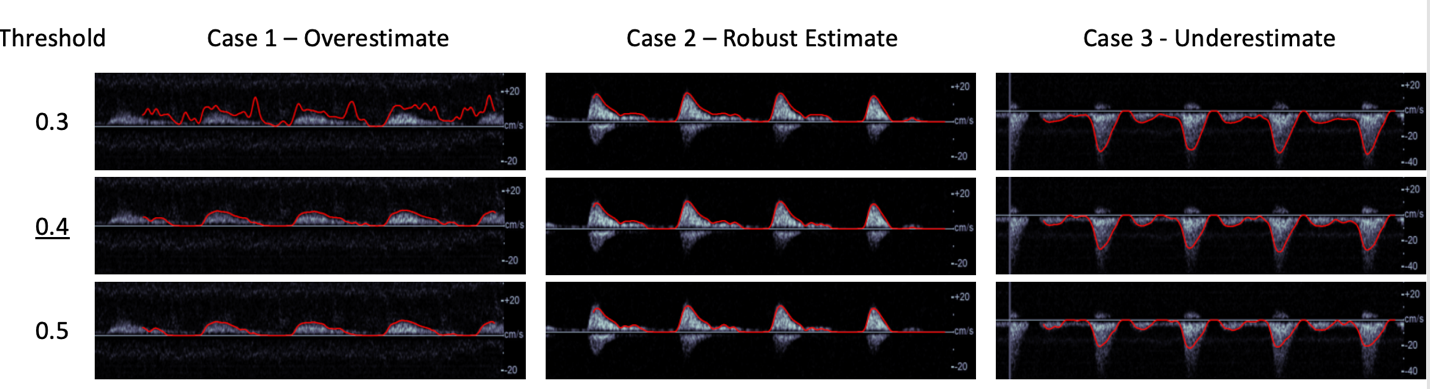


**Figure S-2.** Effect of brightness threshold on Doppler waveform extraction. Case 1: a 30 % threshold admits background speckle, overestimating velocity. Case 2: waveform remains stable across thresholds. Case 3: higher thresholds (40 % and 50 %) truncate the apex, underestimating velocity.

**Step 3 – Spectral smoothing.**
A Gaussian filter was applied to the extracted spectral curve to suppress residual noise and enhance waveform clarity. The Gaussian kernel performs a weighted convolution whose coefficients follow a normal distribution, thereby functioning as a low-pass filter that attenuates high-frequency noise while preserving the fundamental morphology and amplitude of the Doppler waveform. As illustrated in Figure S-3, gaussian smoothing generates a noise-reduced, continuous envelope, thereby enabling precise estimation of PSV, EDV, mean velocity, and other Doppler parameters.


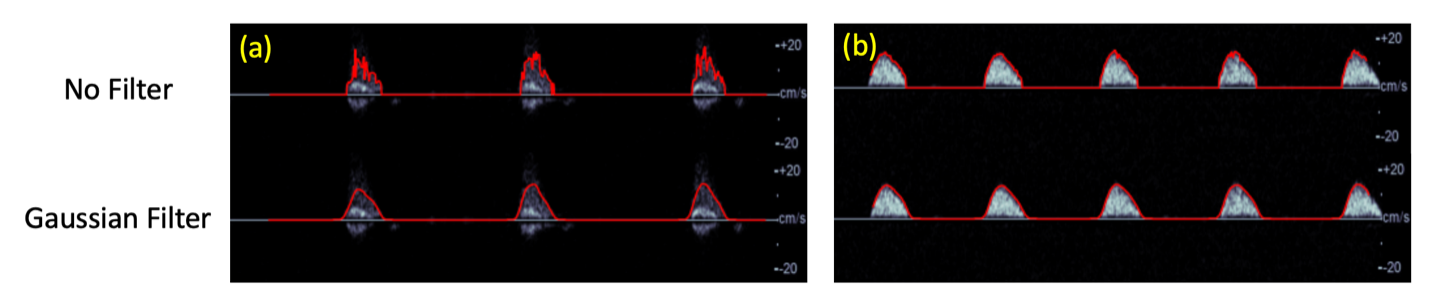


**Figure S-3.** Effect of Gaussian filtering on extracted waveforms: (a) demonstrates pronounced smoothing (most cases), whereas panel (b) shows minimal change.
